# *Breakingtheice*: A protocol for a randomised controlled trial of an internet-based intervention addressing amphetamine-type stimulant use

**DOI:** 10.1186/1471-244X-12-67

**Published:** 2012-06-25

**Authors:** Robert J Tait, Rebecca McKetin, Frances Kay-Lambkin, Kylie Bennett, Ada Tam, Anthony Bennett, Jenny Geddes, Adam Garrick, Helen Christensen, Kathleen M Griffiths

**Affiliations:** 1 Centre for Mental Health Research, Australian National University, Australian Capital Territory, Australia; 2 Centre for Research on Ageing, Health and Wellbeing, Australian National University, Australian Capital Territory, Australia; 3 National Drug and Alcohol Research Centre, University of New South Wales, New South Wales, New South Wales, Australia; 4 Centre for Brain and Mental Health Research, University of Newcastle, Newcastle, New South Wales, Australia; 5 Black Dog Institute, Prince of Wales Hospital, Randwick, New South Wales, Australia

**Keywords:** Amphetamine related disorders, Internet, World wide web, Randomized control trial, Cognitive therapy

## Abstract

**Background:**

The prevalence of amphetamine-type stimulant use is greater than that of opioids and cocaine combined. Currently, there are no approved pharmacotherapy treatments for amphetamine-type stimulant problems, but some face-to-face psychotherapies are of demonstrated effectiveness. However, most treatment services focus on alcohol or opioid disorders, have limited reach and may not appeal to users of amphetamine-type stimulants. Internet interventions have proven to be effective for some substance use problems but none has specifically targeted users of amphetamine-type stimulants.

**Design/method:**

The study will use a randomized controlled trial design to evaluate the effect of an internet intervention for amphetamine-type stimulant problems compared with a waitlist control group. The primary outcome will be assessed as amphetamine-type stimulant use (baseline, 3 and 6 months). Other outcomes measures will include ‘readiness to change’, quality of life, psychological distress (K-10 score), days out of role, poly-drug use, help-seeking intention and help-seeking behavior. The intervention consists of three modules requiring an estimated total completion time of 90 minutes. The content of the modules was adapted from face-to-face clinical techniques based on cognitive behavior therapy and motivation enhancement. The target sample is 160 men and women aged 18 and over who have used amphetamine-type stimulants in the last 3 months.

**Discussion:**

To our knowledge this will be the first randomized controlled trial of an internet intervention specifically developed for users of amphetamine-type stimulants. If successful, the intervention will offer greater reach than conventional therapies and may engage clients who do not generally seek treatment from existing service providers.

**Trial registration:**

Australian and New Zealand Clinical Trials Registry (www.anzctr.org.au/) ACTRN12611000947909

## Background

Amphetamine-type stimulants (ATS) include an array of psychoactive substances, the most commonly used illicit ATS being methamphetamine, amphetamine and ecstasy (3,4-Methylenedioxymethamphetamine) [1,2]. There are now an estimated 35 million ATS users worldwide compared with combined total of 29 million opioid and cocaine consumers [3,4]. The age of initiation of ATS is typically in the mid-late teens, with levels of use often peaking in early adulthood. In Australia, the lifetime prevalence of use is highest among those aged 20-29 years (ecstasy 25%, meth/amphetamine 15%), with the respective 12-month figures being 10% and 6% among the same age group [5].

While the bulk of ATS use is recreational in nature, this population includes a sizable proportion of people who become dependent and experience chronic debilitating health problems as a result of their use of ATS [6]. Major health concerns include paranoia, aggression, increased risk of stroke and cardiovascular pathology [7]. Dependence on ATS, like dependence on any drug, is typically associated with a reduction in quality of life, premature mortality and elevated levels of crime [8]. This is particularly the case with the use of more potent ATS, such as crystalline methamphetamine, and with more efficient routes of administration (e.g., smoking or injecting) [9]. Reducing the impact of ATS use requires the simultaneous development of effective management strategies and methods to improve the uptake and accessibility of treatments for this population. The spectrum of ATS use will also need to be considered, from irregular use through to stimulant use disorders (abuse, dependence) [10].

Despite the widespread use of ATS, a systematic review concluded that, to date, there are insufficient data to support the use of pharmacotherapies such as fluoxetine, amlodipine, imipramine and desipramine in the treatment of amphetamine abuse and dependence [11]. However, research continues to assess other potential agents in the treatment of stimulant abuse [12,13]. A review of psychosocial treatments for methamphetamine dependence reported that the intensive application of psychological interventions (e.g., contingency management, cognitive behavior therapy (CBT), motivational interviewing) can result in a moderate reduction in stimulant use [14]. Brief cognitive behavioral interventions, of up to four sessions duration, have also been shown in previous research to be associated with significant reductions in amphetamine use and significantly greater likelihood of abstinence than controls [15]. However, these types of structured psychological interventions are not widely implemented in community-based treatments for drug use, and ATS users seeking help from traditional drug and alcohol services frequently report their needs are not being met [10]. For example, among a sample of methamphetamine users in Queensland, Australia, the majority felt that more information about methamphetamine use should be available and more accessible outside treatment services and business hours [16]. In particular, respondents reported that needle and syringe programs, methadone maintenance programs and outpatient counseling should not be co-located, as doing so is viewed as a key barrier to treatment access. The need to develop appropriate, novel treatments that are well accepted by ATS communities is clear, including options for accessing treatments outside mainstream specialist treatment services [17].

Given that psychological treatments can reduce stimulant use [15,18], there is the potential to develop internet-based treatments for ATS users, with many internet-based CBT treatment packages currently accessible via the world wide web [19]. The critical advantage offered by web-based interventions for mental health and substance use problems is that they have the potential to greatly reduce key barriers to obtaining treatment, in particular, stigma (given the anonymous access), cost (with free treatment), reach (potentially worldwide coverage and 24 hour access), especially for those living outside of metropolitan areas [20,21]. This is important in the context of ATS use, not only because of the number of people potentially requiring treatment but because surveys show that stimulant users are reluctant to seek treatment from existing drug treatment services as most are tailored toward clients with opiate or alcohol problems [1,22]. Furthermore, for those with addictive disorders, access to treatment ‘24/7’ may be a particularly important benefit of internet services, allowing the potential to capitalize on times of high motivation to change behavior and access to resources when the risk of relapse is high.

Internet-based interventions have already been demonstrated as effective for the treatment of depression and a range of anxiety disorders [23]. With respect to substance use, web-interventions have been effective in reducing problematic use of alcohol in young adults [24] and in reducing cannabis use via school-based interventions that involve on-line materials [25]. Brief internet interventions have been shown to reduce drug use and ‘high-risk’ behavior in human immunodeficiency virus (HIV) positive drug users [26] and a computerized intervention using combined CBT and motivational interviewing (MI) has been reported to reduce cannabis and alcohol use among those with co-morbid major depression [27]. However, the current authors are not aware of any evaluated internet based interventions for the use of ATS.

The objective of the study was to adapt existing face-to-face psychological approaches (i.e., CBT and MI) for delivery via the internet to reduce the use of ATS and associated problems plus improve motivation to reduce ATS use, with assessment of effectiveness at 3 and 6 months post-intervention.

## Method

### Design

A two-group randomized controlled trial will be used. The intervention group will receive an internet intervention consisting of three modules (detailed below). The wait-list control group will undertake the same assessments as the intervention group, with access to the intervention site at 6 months post enrolment. In addition, all participants will be provided with contact details for emergency services.

### Sample

Participants will be recruited via a variety of sources: youth magazines, web-sites, social networking sites, universities and clinics. The inclusion criteria are: resident in Australia, aged 18 years or older and use of amphetamine type stimulants (e.g. meth/amphetamine, ecstasy, non-medical use of prescription stimulants) in the last 3 months. Furthermore, participants need to have access to the internet, a valid e-mail address, telephone access and to provide informed consent. The exclusion criteria are: currently receiving any treatment for stimulant abuse/dependence, currently receiving pharmacotherapy for any substance use disorder (e.g. methadone, naltrexone, buprenorphine) except nicotine replacement therapy; self-reported lifetime diagnosis with schizophrenia, schizoaffective, or bipolar disorder.

### Procedure

**Figure 1:**
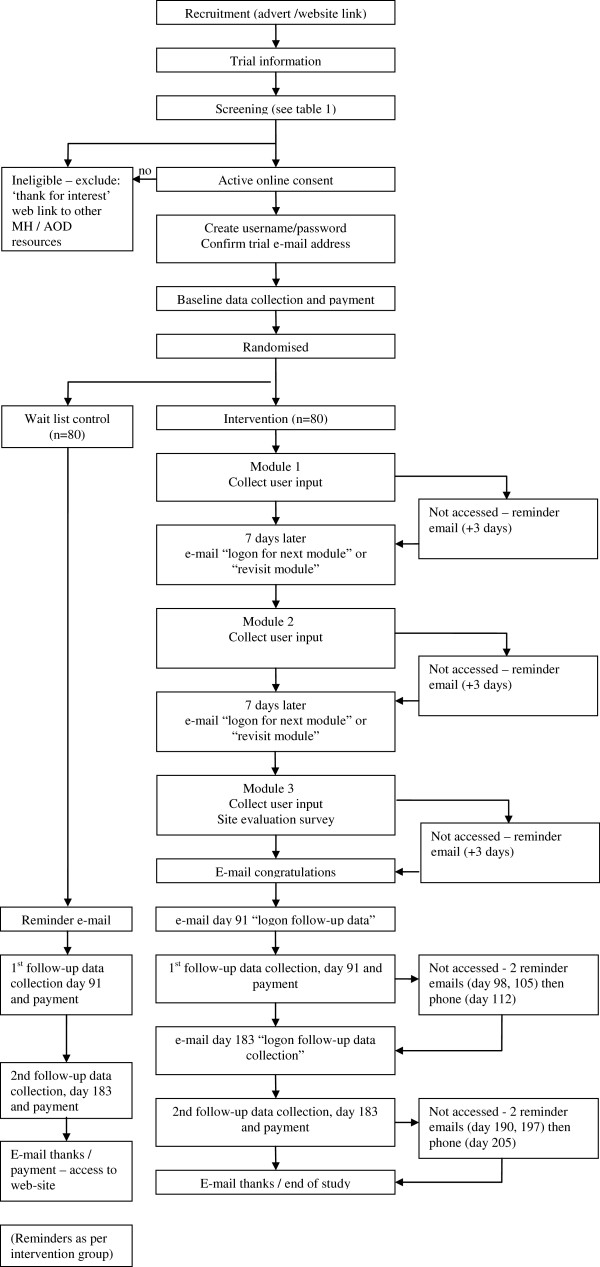
Participant flow through the study.

Enrolment and screening will be undertaken via the internet. Advertisements will direct participants to the study web address. First, participants who visit the study website will be given information on the project and their eligibility assessed by means of a series of online questions. Based on their answers, participants who are not eligible for the study will be given information about other potentially useful websites and resources (e.g. mental health, alcohol or other drugs websites and helplines). Participants who are eligible for inclusion will be asked to provide active consent by ‘clicking’ on a box for each element of the consent form in order to enroll in the study. Figure1 illustrates the flow of participants through the study. Participants will be asked to provide an e-mail address that will be verified via a personalized link in an automated email. This link will take participants to the study site where they will set their own username and password. Participants will then be directed to an online baseline survey before being randomized to study groups. The randomization process will be fully automated with permuted blocks of four and will be implemented within the program.

Participants who are assigned to the intervention group will then be provided with immediate access to the first module of the intervention. These participants will be advised to allow 1 week between modules, but are allowed to proceed at their own pace. Participants must visit each page of a module in sequence to complete the module and thus obtain access to the next one. The intervention group will receive emails at seven day intervals after the commencement of the trial period, either asking them to start the next module, if they have not already commenced that module, or inviting them to revisit the site. A further e-mail will be sent to those who do not start the current module at 3 days after the module was scheduled for commencement. Participants will be invited via e-mail to complete follow-up assessment at 3 and 6 months (91 and 183 days) post-randomization with a further two reminder emails at 7 day intervals if required, followed by a telephone call(s) for participants who do not respond to the emails. Participants will receive AU$20 for baseline assessment and for each follow-up questionnaire that they complete, paid as either on-line vouchers or posted cheques.

The Australian National University Human Research Ethics committee approved the study, which is also registered with the Australian and New Zealand Clinical Trials Registry (www.anzctr.org.au/) ACTRN12611000947909.

### Measures

Both the primary and secondary outcome measures will be assessed at baseline, 3 and 6 month. The primary outcome measure will be ATS use, assessed using the World Health Organization Alcohol, Smoking, Substance Involvement Screening Test (ASSIST) [28,29]. The ASSIST first assesses lifetime use of nine categories of drugs (i.e. tobacco, alcohol, cannabis, cocaine, ATS, inhalants, sedatives, hallucinogens, opioids and other). Then, for any drug identified by the lifetime question, data relating to the last 3 months is collected on: frequency of use, cravings, problems (health, social legal or financial), and failure to fulfill roles. Next, the ASSIST ascertains if a friend or relative has ever expressed concern about their drug use and if the person has ever tried and failed to control their drug use. Finally, injection of drugs is assessed. The standard ASSIST scoring algorithm will be used to generate a score related to use of ATS [28].

The secondary outcomes will be: (a) health-seeking intention and help-seeking behavior measured with the general help-seeking questionnaire (GHSQ) [30] and the actual help-seeking questionnaire (AHSQ) [31,32]; (b) readiness to change assessed using a modified version of the Readiness to Change Questionnaire (RTCQ) [33] referencing ATS rather than alcohol; (c) psychological distress assessed using the Kessler 10 [34]; (d) poly-drug use measured by the ASSIST [28]; (e) days out of role [35]; and (f) quality of life assessed by the European Health Interview Survey (EUROHIS) Quality of Life scale [36]. The baseline measures include demographic information (e.g., age, sex, marital status), drug use history (age of first use of ATS), and severity of dependence assessed using the Severity of Dependence Scale [37].

Both the GHSQ and AHSQ are designed to be modified to reflect the condition under investigation and relevant potential sources of help [32]. The condition investigated will be help-seeking for ‘a problem with stimulant drug use’, with additions to the existing list of potential sources of help including “drug information services (e.g. internet, telephone)” and “specialist drug services (e.g. in-person services)”. We will also modify examples such as “school counselor” or “teacher” to reflect the target age group (“counselor” or “lecturer”). Item scores will be summed on the two measures. The RTCQ has 12 items, with four questions relating to each of the stages: ‘pre-contemplation’; ‘contemplation’; and ‘action’. The five point Likert scales will be summed to obtain scores on each stage, with participants designated to their highest scoring stage, or in the event to tied scores, the higher stage [33]. The K10 total score (maximum 50) will be used to quantify psychological distress at each time point [34]. Poly-drug use will be defined as the sum of ASSIST classes of drugs endorsed for the past 3 months [29]. ‘Days out of role’ will be derived from Kessler’s questions but referencing “ATS drug use (e.g. methamphetamine, ecstasy, ice) “rather than “depression” [35] and quality of life will be indexed as total EUROHIS Quality of Life scores [36].

In addition, immediately after finishing the third module, the intervention group will evaluate the intervention content and study website. Satisfaction with the intervention plus perceived benefits and negative aspects will be assessed with a measure developed for the *Wellbeing* study [38] but modified to reference “drug use” rather than “depression”. Descriptive measures of completion (e.g. number of modules and subsequently, follow-up surveys) will be collected together with scores on the Internet Intervention Adherence Questionnaire – 16 items evaluating barriers to internet treatment [39]. Table 1 provides a summary of the measures assessed at each time point.

### Estimation of the expected effect sizes and sample size

With three time points (baseline, 3 and 6 months) the study is designed to detect an effect of *f* = .27. This requires 60 people per group: to allow for attrition of 20% by 6 months we will recruit 80 people per group. The development study for the ASSIST reported that in an Australian sample of stimulant users, there was a significant group by time interaction for those who received a brief intervention compared to controls (with 64 and 65 people respectively per group), which had a power of 84% to detect this effect [28]. Other brief in-person motivational interventions have achieved moderate sized effects on drug use measures (*d* = .63-.45) [40], so the effect size postulated for this study is plausible.

### Analysis

The primary analysis will be on an intention-to-treat basis, using a mixed model repeated measures (MMRM) approach. This overcomes many of the limitations of traditional repeated measures analysis of variance, in that it uses all available data without requiring substitution or estimation of missing values to avoid the exclusion of cases with non-complete data and does not assume homogeneity of correlations over waves of measurement [41]. The effect of the intervention will be assessed by an analysis of a time by group interaction. A sensitivity analysis will be conducted using multiple imputation of missing data. Categorical outcome measures will be evaluated using non-linear mixed models.

### Content of modules

The theoretical approach reflected in the intervention modules is motivational enhancement with use of CBT based methods. The intervention draws on guidelines for face-to-face treatment of amphetamine use [42]. The underpinning philosophy is that of harm minimization, with participants free to decide on the most appropriate goals for themselves, including quitting completely, reducing their drug use, taking a break from use, or using in a less hazardous manner.

**Figure 2:**
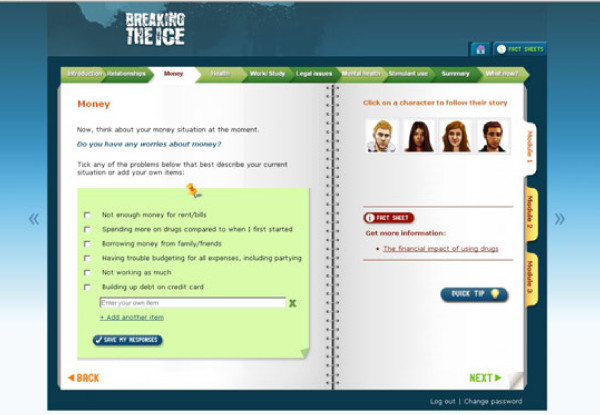
Screen shot from module 1 – money problems.

The first module examines the key problem areas on which the use of ATS typically impacts. Participants either select from a menu of items for each problem area or record relevant problems that they have incurred. The areas are: relationships with family and friends, health, finances, work/study, legal issues, mental health, and specific drug use problems (see Figure2). Each page of the intervention features four characters, with a developing storyline for each character that involves a problem relevant to that page; for example, problems that have arisen in a work setting. The final page provides a summary of the problems that the participant has endorsed and guides the participant to generate a ‘map’ of the interconnections between problems.

**Figure 3:**
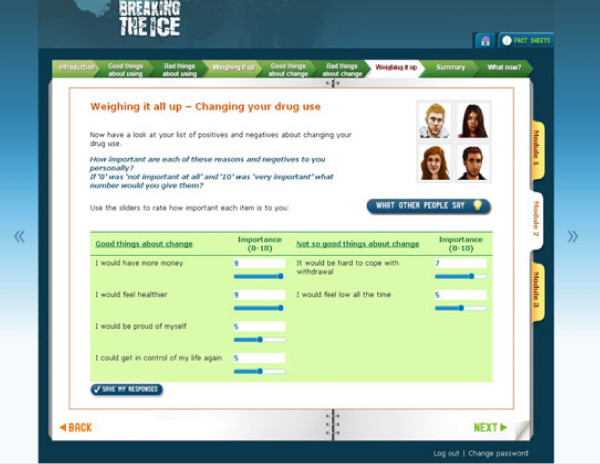
Screen shot from module 2 – weighing up the pros and cons of changing drug use.

Module two asks participants to think about the pros and cons of their use of stimulants and the good and bad things related to changing their behavior, an approach based on the model of Miller and Rollnick [43]. To aid in their ‘decision balance’ for each good or bad element that they select, participants are asked to rate its importance on a 1-10 scale (see Figure3).

The third module focuses on behavioral change including techniques such as setting clearly specified goals, actions on specific dates, strategies to help with controlling and overcoming cravings, refusal skills, managing a ‘slip’, and an action plan to deal with high risk situations.

### Safety & security

The intervention is not intended to provide emergency treatment, and the participant information sheet and website for the study provide emergency contact numbers for appropriate providers. If distressed participants contact the research team, the Centre for Mental Health Research has a written protocol to guide members of staff in handling the situation. All user data collected via the study website and the online intervention are securely stored according to a written protocol, and access to data is limited to research staff who are named on the ethics protocol [44].

## Discussion

To the best of our knowledge, this is the first internet intervention developed specifically for users of ATS. Outcomes will be evaluated against critical measures, including changes in the use of ATS, improved mental wellbeing and quality of life. The randomized design and 6 month follow-up will allow firm conclusions to be drawn and, if successful, will justify the dissemination of the site into an effectiveness trial, i.e. under ‘real world’ conditions, and with evaluation to 12 months. There is already evidence that it is feasible and effective to transition internet interventions for substance abuse from randomized trials into open access sites to provide resources to the general community, at least in the case of cessation of smoking [45].

There are a number of ethical issues that are common to internet interventions. Firstly, for ethical reasons the minimum age for participants is 18 years. Although participants are explicitly asked to confirm that they are aged 18 years or older, it will not be possible to ensure that all participants are adults [46]. Secondly, since participants are recruited via an online mechanism, the extent that consent is ‘informed’ is not as transparent compared with face-to-face recruitment. However, the consent process includes multiple tick boxes covering critical issues and provides direct contact details of the chief investigator, as recommended [47]. Third, it is not possible to gauge if participants have become distressed by the content or activities of the intervention and provide clinical support. Therefore, the information sheet and resources in the trial web-site contain contact details for relevant agencies. Fourth, the study uses a waitlist control rather than an ‘attention control’, which means that exposure to the experimental setting could account for any changes observed, rather than the content of the modules.

It has been suggested that internet interventions incur a greater level of attrition than face-to-face interventions which threatens their internal validity [48]. The study has been designed to be ‘front-loaded’ with screening and baseline questionnaires applied before randomization and entry into the study; this may encourage early drop out by those with low motivation [48], but may limit subsequent generalization to more motivated participants. The use of MMRM analysis maximizes the use of available data collected during the study to minimize biases associated with attrition. A particular concern for internet interventions in the substance use domain is that of self-report of drug use without the potential to use biochemical verification (i.e. urine samples) as often used in face-to-face intervention trials. Nevertheless, there are data to support the reliability and validity of self-reported illicit drug use: indeed self-reports can reveal a greater extent of drug use than biochemical screening, with most illicit drugs only detectable for a short period [49].

If the intervention is effective, it will offer advantages over conventional, face-to-face interventions in that internet interventions can provide treatment virtually nationwide. In Australia, 80% of the population already has access to the internet at home with further access provided by public facilities such as libraries [50]. There is also the potential for access to the site by an increasing proportion of the population of the world. Further, the anonymous nature of the intervention may reach participants who are reluctant to engage with traditional drug treatment services due to fear of stigmatization or the belief that the services offered are not appropriate for those with ATS related problems rather than alcohol or opioid problems.

## Status of the trial

Recruitment is planned to commence in mid-2012

## Abbreviations

AHSQ, Actual help-seeking questionnaire; ASSIST, Alcohol, Smoking, Substance Involvement Screening Test; ATS, Amphetamine-type stimulants; CBT, Cognitive behaviour therapy; EUROHIS, European Health Interview Survey; GHSQ, General help-seeking questionnaire; K-10, Kessler 10 questionnaire; MI, Motivational interviewing; MMRM, Mixed model repeated measures; RTHQ, Readiness to Change Questionnaire.

## Competing interests

None of the authors has any competing interests with respect to the study.

## Authors’ contributions

The study was designed by RT, RM, FK-L, KB, AB, HC and KG. The intervention content was developed by FK-L, JG, AG, KB and AT, with web implementation by AT, AB and KB. The initial draft manuscript was by RT and RM. Funding was obtained by KG, RT, RM, FK-L and HC. All authors revised and contributed to the development of the manuscript and gave approval for the final version to be published. All authors read and approved the final manuscript.
